# A Linked Data Mosaic for Policy-Relevant Research on Science and Innovation: Value, Transparency, Rigor, and Community

**DOI:** 10.1162/99608f92.1e23fb3f

**Published:** 2022-04-28

**Authors:** Wan-Ying Chang, Maryah Garner, Jodi Basner, Bruce Weinberg, Jason Owen-Smith

**Affiliations:** 1 National Center for Science and Engineering Statistics, National Science Foundation, Alexandria, Virginia, United States of America; 2 Coleridge Initiative, University of Maryland, New York University, and University of Chicago, Brooklyn, New York, United States of America; 3 Clarivate Analytics, Rockville, Maryland, United States of America; 4 Department of Economics, College of Arts and Sciences, Ohio State University, Columbus, Ohio, United States of America; IZA Institute of Labor Economics, Deutsche Post Foundation and the University of Bonn, Bonn, Germany; National Bureau of Economic Research, Cambridge, Massachusetts, United States of America; 5 Department of Sociology, Institute for Research on Innovation and Science, University of Michigan, Ann Arbor, Michigan, United States of America

**Keywords:** science, innovation, community, linkage, graduate education

## Abstract

This article presents a new framework for realizing the value of linked data understood as a strategic asset and increasingly necessary form of infrastructure for policy-making and research in many domains. We outline a framework, the ‘data mosaic’ approach, which combines socio-organizational and technical aspects. After demonstrating the value of linked data, we highlight key concepts and dangers for community-developed data infrastructures. We concretize the framework in the context of work on science and innovation generally. Next we consider how a new partnership to link federal survey data, university data, and a range of public and proprietary data represents a concrete step toward building and sustaining a valuable data mosaic. We discuss technical issues surrounding linked data but emphasize that linking data involves addressing the varied concerns of wide-ranging data holders, including privacy, confidentiality, and security, as well as ensuring that all parties receive value from participating. The core of successful data mosaic projects, we contend, is as much institutional and organizational as it is technical. As such, sustained efforts to fully engage and develop diverse, innovative communities are essential.

## 1. Introduction

Over the last two decades, computational and data revolutions have dramatically shifted the landscape for fundamental research and policy analysis. Recent changes in law, such as the Foundations for Evidence-Policy Act of 2018 (2019), and administrative efforts, such as the Federal Data Strategy, highlight a transformation in how the U.S. government views data as a strategic asset. Recent executive orders emphasize that far from being a neutral commodity, data—its development, characteristics, accessibility, and use—are key to addressing persistent disparities and ensuring equality of opportunity in the United States (Exec. Order No. 13985, 2021). All these policy developments (and parallel trends in industry and academe) highlight that in today’s world, data are a necessary form of infrastructure that require strategic development, the success of which is implicated in nearly every aspect of government and civil society.

But what does it mean to consider data a strategic asset on par with other, more tangible forms of infrastructure? How can a 21st-century data infrastructure that protects privacy, ensures equity, and transparently enables rigorous, timely research and analysis across a wide range of topics be developed, maintained, and used? We propose that the answers to these questions are as much social and institutional as they are technical. This article seeks to develop a framework for creating and maintaining such an infrastructure. We identify the social and institutional conditions under which large-scale, often restricted data might be productively and sustainably integrated to support and improve fundamental and evidence-based policy research in multiple domains.

Substantial linked data assets are not new. The intellectual and analytic value of analyses that draw together data from multiple sources is not news. However, we argue that current models do not realize the full value or permit the same flexibility as the ‘data mosaic’ structure that we propose here. One prominent approach tends toward a centralized, hierarchical form of organization that makes the timelines, goals, and needs of a small number of near monopolies preeminent in the design, protection, and use of data infrastructures. This structure can work in some contexts but is insufficient to realize the vision of contemporary evidence-based policy research in many important cases, especially when data resources are widely held by different, co-equal custodians.

In such cases, a hierarchical model often resolves to attaching one or more ‘child’ data sets to a ‘parent’ data set where the child data sets are typically used in conjunction with the parent, but not with each other (i.e., not independently of the parent data set). Often, thin slices of such integrated data can be made available for public use with few or no restrictions, allowing broad, decentralized work by many users. However, such data releases generally lack the features necessary to support a comprehensive, equitable, general-purpose data architecture. A second approach involves attaching thin slices of data from one or more child data sets to a parent data set to answer a specific question. Such work may be artisanal and hard to replicate, and any resulting integrated data generally lack the features necessary to support a broad, equitable, general-purpose data architecture.

A new approach is necessary because the value of large-scale data infrastructure is manifest. Lengthy traditions of research in sociology, organizational theory, and economics can inform an organizational framework and set of principles that avoid the pitfalls of too much centralization on the one hand and too little coordination on the other.

We propose that realizing the full value of linked data for both fundamental research and policy analysis requires a network form of organization and a set of practices to promote generalized exchange among stakeholders with very different goals, who face varied, sometimes contradictory legal, ethical, and proprietary restrictions. Mindful efforts to organize and sustain diverse, innovative communities that build, protect, and use data are essential.

There has been much work done on the statistical algorithms used for data integration (Baldwin et al., 1987; Batini & Scannapieca, 2006; Christen, 2012; Herzog et al., 2007; Talburt, 2011; Winkler & Newcombe, 1989), but to our knowledge, this is the first article that focuses on the network form of organization that is essential to cooperative long-term partnerships necessary to establish a linked data mosaic. Our argument centers on the conditions necessary for, and the substantial value of, linked data mosaics. We take such mosaics to have several key features. They are: (1) collectively designed and collaboratively integrated data architectures that (2) support coherent, productive, accessible, and equitable data infrastructures and (3) deeply integrate a wide range of co-equal data sources (4) through the work of a diverse, decentralized but interdependent community. We distinguish data mosaics from hierarchical data infrastructures that subordinate partners, users, and data to the needs of a powerful, central authority. We also distinguish data mosaics from decentralized systems that make carefully curated, but often thin slices of data broadly available to users who have little input in their creation but who creatively integrate them with often laboriously collected data sets tuned to address a particular topic or question.

In what follows, section 2 presents a stylized model of the value of linked data mosaics. Section 3 summarizes the key features of an innovative data science community organized as a network form of governance. We attend particularly to the dangers of relying on network communities to develop and sustain reliable, secure, and equitable data infrastructures. Close focus on a particular case, where a data mosaic is valuable —establishing the value of science through the people who conduct it—concretizes our more abstract conceptual claims. We lay out that context in section 4. Section 5 sketches the two organizations that are at the heart of our work: the Institute for Research on Innovation & Science (IRIS) and the National Center for Science and Engineering Statistics (NCSES). Section 6 discusses the issues that have arisen in linking these data sets. Section 7 presents initial linkage results that highlight the value of our integration of three types of data: transaction-level administrative data on research funding, national survey data on the population of doctoral recipients, and bibliometric data from scientific publications. Section 8 concludes by laying out lessons that can be drawn from our partnership.

## 2. Value of the Network Form

We begin with a toy model to make the general case for linked data, especially the value of linked data in a mosaic. In discussing linked data, it is important to emphasize that to be fruitfully linked two data sets must be relatable (i.e., cover common units and have common identifiers). To see the power of linked data, we posit the relationship *Y* = *Q*^*D*^, where *Y* denotes the knowledge that can be produced by research using given data; *Q* is a constant that denotes the number of questions that can be answered per distinct data element available; and *D* denotes the number of distinct data elements that are available. Consider an area where each data element can be used to study two questions (i.e., *Q* = 2). If there are two data elements available (*D* = 2), then four questions can be answered, but if we increase the number of data elements to three (*D* = 3), then eight questions can be answered, and so forth. Thus, we posit that the value of linked data is that it generates exponential growth by adding in more distinct data elements.

A few points are worth clarifying. First, by distinct data elements, we mean data elements that do not overlap substantially, such as measures of hours worked in one week and an hourly wage, instead of two measures of hours worked in two, unexceptional, consecutive weeks. Second, the constant Q in our model should not be taken as an immutable, universal constant like Planck’s constant, but rather one that is likely to vary across data sets based on their data elements. Indeed, different combinations of data sets are likely to have different values of *Q* depending on their complementary data elements and possibilities for linkage. Thus, our use of a given value of *Q* should be viewed as a heuristic rather than definitive. Second, we note that the final value of Y depends not just on the characteristics of data sets but also on the conditions under which they were combined, rules for their accessibility and use, and the characteristics of their potential users. In short, the potential value of linked data architectures is determined by the characteristics of their component pieces, but also by the larger socio-organizational contexts in which they are created, sustained, and used. Third, from the perspective of economic industrial organization, exponential value or any form of increasing returns generates the potential for a monopoly in industry as well as in government or research, a danger that has to be managed and for which we propose governance mechanisms.

**Figure 1.**
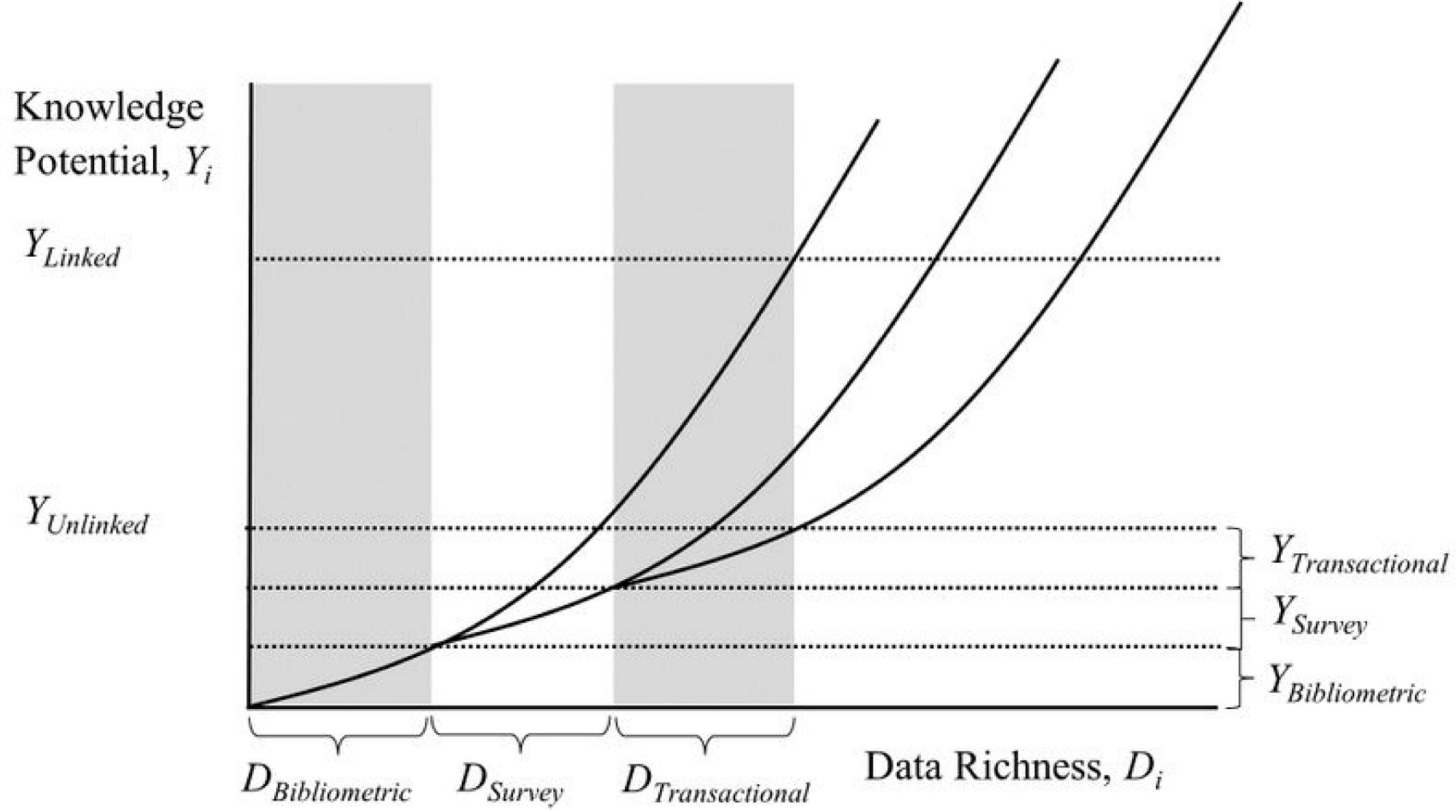
Power of linked data. The figure illustrates the case where there are three sets of distinct data elements, bibliometric, survey, and transactional. If these three data elements are not linked, it is possible to produce knowledge of *Y*_*Unlinked*_, but if these data elements are linked, it is possible to produce considerably more knowledge, *Y*_*Linked*_.

Figure 1 illustrates the case where there are three sets of distinct data elements, bibliometric, survey, and transactional, which correspond very broadly to some of the main classes of data relevant for our illustrative example, innovation. The specific types factor into the discussion, but are less critical for the moment. What is critical is that each represents distinct data elements from the others. If these three types of data are not linked and we assume that they each comprise the same number of data elements (*D*) of comparable utility (*Q*), it is possible to produce knowledge of *Y*_*Unlinked*_ = 3*Q*^*D*^, but if these data elements are fully linked and interoperable in mosaic fashion, it is possible to produce considerably more knowledge, *Y*_*Linked*_ = *Q*^3*D*^. Intuitively, the combination of different data allows for an exponential expansion in opportunities compared to unlinked data because it increases *D* dramatically from a very high initial level, greatly increasing potential knowledge. More concretely, any of the explanatory variables in a given data set can be used to analyze any of the outcome variables present in any of the others. Further, it is likely that some of the ‘explanatory’ variables in one data set will be valuable as outcome variables when linked to another data set and vice versa.

We have already mentioned two common ways of linking data that are distinct from a data mosaic, in which the data sets are linking on multiple keys and where each pair of data sets can be used in conjunction with or independently of the others because integration work builds rich linkages among all relevant pairs of data sets. Contrast this case to the two stylized alternative models: decentralization and hierarchy. In the former, individual researchers might construct a data set suitable to answer their particular questions and then draw on a publicly available, already curated data set to compute additional variables (e.g., as explanatory variables or instruments) they then integrate with their data to address one or more concrete research questions. An example in the case of innovation might be to merge some data on the institutions at which researchers work drawn from publicly available sources such as the Higher Education Research and Development (HERD) survey into proprietary bibliometric data to provide contextual variables based on author affiliations. In this decentralized case, we expect there to be some gain from integration, but we anticipate lower values of *Q* and *D* because the ability to combine data sets is relatively limited as are the units of analysis that might be examined.

More importantly, perhaps, this kind of decentralized, artisanal approach to data integration might allow replication and potentially some extension of research depending on individual data-sharing practices, but would not result in a broadly accessible and expandable resource and would not, without significant further work, represent a component of a larger integrated data architecture. As a result, the range of questions that this kind of decentralized approach might be able to address and the knowledge that might be produced through their answers will be relatively limited.

A second approach mentioned earlier is hierarchical, in which one data set is the parent and other child data sets are linked to it. For instance, restricted administrative data from many organizations might be linked to a restricted federal data asset. Here the value hinges on whether the child data sets are integrated with each other and on whether the links between them can be accessed (in terms of data architecture and data use provisions) without the parent data set. If the child data sets were themselves integrated and usable with each other and without need to rely on the parent data set or adhere to its rules for access and use, then the gains might be comparable to those of a linked data mosaic, provided the data are accessible by the relevant community and their custodians are responsive to community needs. If, as is more typical, the various child data sets are only linked indirectly through each’s integration with the parent—a situation that would not permit their use independent of the parent data set —the potential knowledge available from combining the parent data set with each child data set would be *Y* = *Q*^2*D*^ and the gain from combining the parent data set with two child data sets would be *Y* = 2*Q*^2*D*^. This is considerable, but for reasonable values of D unlikely to produce the same gains as a linked data mosaic, where the potential knowledge available from fully integrating the three data sets is *Y* = *Q*^3*D*^.

The network form, or linked data mosaic, maximizes the potential uses of each data set through linkage because it allows many possible integration strategies and levels of analysis to be explored in use by the relevant community members who seek to develop them for their own purposes. The linkage dimensions can be defined using all possible analysis units, or all identifiers that exist in the data. For instance, in the Universities Measuring the EffecTs of Research on Innovation, Competitiveness and Science (UMETRICS) case we consider in more detail below, a transaction from one project (payer) can be linked for all payees (person or vendor). Moreover, when a new data source enters the architecture through linkage to an already integrated component, it does not merely become available for analysis using the data set to which it is directly linked. Instead, additional possibilities are opened because the new data can be indirectly connected to all other data sets that are ‘reachable’ because of their integration in the larger architecture, which can also be conceptualized as a network where data sets themselves are the nodes. In those terms, part of the power of a mosaic approach is that new nodes can potentially be linked to other existing related nodes in the network because the existing nodes are all already integrated (or at least integratable) with each other. While we have emphasized the potential value of linking data sets, there are many factors to consider when exploring whether to link a pair of linkable data sets. Technical feasibility is often not the determining factor. Rather, the benefits of the linkage must outweigh the costs. And, as we argue below, the community-building component is a major benefit in addition to the connected data itself and a way of lowering the cost of linkage.

A separate, slightly more technical advantage of linked data, which is not covered in the figure but potentially quite important in practice, is that a large portion of data construction involves algorithmically cleaning and linking data sets and, while adding data sets increases the amount of linkage and cleaning required, it also greatly increases the data resources available by providing multiple angles on the same data elements. And we speculate that in most cases the gains from greater visibility exceed the complexity of more links.

## 3. Features of the Network Form of Data Integration

Realizing the kinds of synergies our conceptual model highlights requires social and organizational integration as well. If we are to avoid the twin poles of hierarchical, centralized data assets and uncoordinated, decentralized fields while ensuring that participants gain something of value for their work, we require a new model for the construction and maintenance of data mosaics. A conceptual toolkit drawn from sociological, organizational, and network theory can help us solve this problem. Here we focus on the social aspects, briefly introduce two key concepts (network forms of organization and generalized exchange), highlight a few of their features, and identify some points of possible failure. We can then outline the essential components of an innovative learning community built around integrated data and its effective use, which we illustrate with examples drawn from a data-integration relationship between IRIS and NCSES. That partnership highlights both the technical and the socio-institutional features required to start building the kind of generative community that can maintain and expand a data mosaic of the sort we describe. This community matches a cohesive network of interdependent data custodians and researchers (a generative community) with a deeply integrated network of co-equal data sets (a data mosaic) that can be used by many community members in a wide variety of combinations (a data infrastructure suitable as a strategic asset for policy and research in multiple domains).

Generative communities are fickle things. Their existence implies a degree of trust and affinity among their members, often anchored on some productive form of interdependence enforced by reputational or other social means. For voluntary communities to work, membership must provide something of value that participants could not realize solely with their own skills and resources. That value must offer sufficient inducement to defer some of their individual interests and some of their autonomy in favor of the community’s health. It must also lead them to stick and stay in the face of the inevitable problems and conflicts that accompany any complex, collaborative endeavor. Communities are defined in part by a willingness to exercise voice in the face of those challenges rather than exit (Hirschman, 2007). These features are particularly hard to develop when communities must draw from diverse groups whose cultures, priorities, and constraints are at least somewhat alien to one another. They are harder to sustain when participants and their interests are at least somewhat inimical. Both things are true of the diverse communities that would be needed to support the national data mosaic we envision.

Several questions follow: (1) what kinds of relationships create and sustain the positive social and affective features of community? (2) what kind of organizational arrangements can support and sustain such relationships? and (3) what dangers must we guard against in cultivating both?

### Question 1. What kinds of relationships?

Voluntary communities that one must choose to join and work to help maintain at some cost to oneself are forged from interdependent goals and value. At the heart of both things is a peculiar social relationship, exchange. Some types of exchange relationships, for instance between strangers in market contexts, are relatively socially thin. Buying an apple from a stranger at a farmer’s market while visiting a distant town requires a lot of formal institutional infrastructure (such as enforceable property rights) (North, 1993) but relatively little social embeddedness (Granovetter, 1985). It is not necessary to have any kind of personal relationship or any degree of commonality or fellow-feeling with the person who sells one a snack. Other types of exchange, like gift giving, or sharing of valuable and costly-to-transfer private information, are more socially complicated (Blau, 1964; Homans, 1974) and require deeper social support. The problems are compounded when exchanges carry significant risk or might be perceived as illegitimate (Healy, 2006).

The kinds of resource exchanges—of access to restricted, complicated data; of expertise and skill—necessary to vibrant data science communities have much of this character. Likewise, the conditions under which relationships must work—under time pressure; when parties’ collaborators might also be rivals pursuing orthogonal or unrelated ends; when there are high scientific and practical stakes—can introduce a substantial degree of risk. Finally, real and important concerns about privacy and security as well as binding legal restrictions raise the possibility that at least some potentially powerful observers might consider the sorts of exchanges we hope to facilitate to be illegitimate.

Under these conditions, we could trust altruism. That has possibilities, but we are realistic enough not to trust it too much. Instead, we should focus on reciprocity, the familiar tit-for-tat of game theory. Reciprocal exchanges are those where one party gives to another with the expectation that similarly valuable resources will be returned. When the shadow of the future is long, that can be sufficient to support the emergence of relatively complex cooperative systems, but it also runs the risk of a downward spiral of defection.

There are two general types of reciprocity. Direct reciprocation happens when one enters an exchange relationship expecting that the specific partner with whom one is exchanging valuable resources will also be the one who gives back. A second form, indirect or generalized exchange, is more diffuse and takes place when one gives to a partner with the expectation of return from a person or organization who was not party to the original exchange. This requires first that there be multiple parties (a community if you will) engaged in similar forms of valuable, reciprocal exchange who are aware of and engaged with one another (Axelrod, 1984 Takahashi, 2000). Generalized exchange is a common form of gift giving among kinship groups (Levi-Strauss, 1955). Someone might offer something of value to a cousin with the expectation that eventually an uncle will return the favor, even if that cousin never does.

This kind of ‘pay it forward’ ethos is subject to real dangers of free-riding or opportunistic exit and thus requires a degree of trust that typically only emerges within a system of ongoing, successful relationships; an exchange network. However, when it works, generalized exchange generates and sustains social solidarity, which encompasses most of the features of generative communities that opened this section (Molm et al., 2007). Kinship clearly won’t do the trick here. Instead, we suggest an organizational scaffold.

### Question 2. What kinds of organizational arrangements?

Network forms of organization exist at a midpoint between markets—the socially thin but institutionally thick contexts for generally short-term, transactional, and armslength exchanges like buying that apple in a far-away farmers market—and hierarchies —the formal bureaucratic structures that characterize large organizations. It seems unlikely that market coordination will result in the kind of community we need. Even if it were possible, internalizing the wide range of data, skills, and expertise necessary to our task in a large bureaucracy would create a dangerous monopoly and might increase security risks by concentrating all the data in a single, presumably fallible organization. We also know that generalized exchange and the benefits it could bring requires a distributed network of interdependent players.

Network forms of organization are more nimble and ‘lighter on their feet’ than bureaucracies and much better at the transfer of complicated, highly tacit information necessary to the sorts of substantive work we would want an innovative data community to accomplish compared to markets (Podolny & Page, 1998; Powell, 1990). They are generally stable enough to create a long shadow of the future and to foster the kinds of trust, informal, reputation-based social control, and forbearance that are necessary in the absence of perfect contracts (Uzzi, 1997). Network forms of organization are common and generally effective in precisely the kinds of diverse, skilled, and creative communities we seek to develop. They are often found in industrial districts, regional technology communities, creative industries, and open-source software projects where rivals on one project may be collaborators on another and where disparate, sometimes conflicting interests and goals are commonplace (Hargadon & Bechky, 2006; O’Mahony & Ferraro, 2007; O’Mahony & Bechky, 2008; Sable & Piore, 1984; Whittington et al., 2009).

Network forms are particularly suited to situations where “the knowledge base is both complex and expanding and the sources of expertise are widely dispersed” (Powell et al., 1996, p. 116). Dispersed, rapidly changing knowledge and complex interdependencies among component parts of innovations characterize this type of governance. Those are also exactly the features of a data science community that seeks to understand, explain, and improve the value of science.

The seed crystals for successful networks have particular characteristics. They are often themselves organizations who anchor communities, convene partners, and make significant, substantive contributions to common projects. Network anchors help to set the technical directions and the tone for their communities (Owen-Smith & Powell, 2004, 2006). Though they lack the authority or power necessary to compel participation or dictate behavior, they exert significant influence by serving as neutral brokers and meeting grounds for participants. Network anchors are most effective when they are active and credible participants in collective efforts and thus can play important roles in fostering new partnerships, engaging new participants, aiding in collaborative problem solving, and helping to build systemwide capabilities so they are in no way disinterested (Owen-Smith, 2018; Powell & Owen-Smith, 2012). Their ability to do all these things is aided by transparency, by rigor, and by the ability to help translate across the different languages and goals of their many partners to help ensure that all find their participation valuable. In doing so, they help provide a basis for the emergence of generalized exchange in a community. As discussed in a later section, a network anchor also performs a valuable service by acting as a neutral broker, collecting and disseminating information that can help identify new partners and smooth their entry into the community. IRIS, which seeks to play just this kind of role in a consortium of major research universities and a growing, interdisciplinary research community, was designed and operates according to these principles.

### Question 3. What are the dangers?

Networks, like markets and hierarchies, can and do fail. Setting aside changes in circumstances rendering their work less valuable and to put it starkly, networks fail “when exchange partners either screw each other or screw up” (Schrank & Whitford, 2011, p. 170). There are instructive differences between screwing your collaborators and screwing up. The former is most often a function of opportunism, “self-interest seeking with guile” (Williamson, 1979, p. 234). The problems of defection, free-riding, self-dealing, and strategic exit from relationships or communities that are dangers of both direct and generalized reciprocal exchange are all examples. While it is often hard to distinguish them in practice, or in the heat of the moment, screwing up is less malignant but no less dangerous. Partners screw up when they lack the competencies or knowledge necessary to successfully pursue complex and difficult collaborative projects. Networks screw up when they stop seeking new information from outside their current orbit and calcify, closing themselves off and becoming unable to identify and adapt to new situations or needs. Such ‘closure’ renders them incapable of the kind of flexibility and responsiveness that help ensure membership in the community is valuable. Beyond natural obsolescence, the problem here is not guile, it is ignorance or a simple competency shortfall. These, along with various forms of opportunism, are the primary dangers for network forms of organization that seek to foster generalized exchange in support of vibrant, innovative learning communities.

In the context of data architectures, innovative communities organized around network forms of governance and oriented toward generalized forms of exchange represent a key difference between what we call a data mosaic and either of the other stylized models for data infrastructure we discuss. This is the case because the potential value of a mosaic derives as much from the conditions under which it is developed, expanded, accessed, and used as from the features of data itself. Linked data architectures are themselves networks where nodes are data sets and ties are defined by links across them. Under conditions where the nodes of such a ‘data network’ are controlled by many different custodians and governed by multiple restrictions, the options for organizing such a network have typically either required centralized control (an analogue of the hierarches that are one pole of the organizational spectrum) or decentralized modes for allowing access to thinner subsets of data that do not run afoul of restrictions on use (an analogue of the markets that represent the other).

In contrast, a vibrant data mosaic that can take full advantage of many different linking assets and levels for many purposes is a network of linked data sets that is grounded in a community infrastructure that neither requires all participants to subordinate their requirements and goals to a single dominant player, nor necessitates the thinning of data to allow them to be used broadly. Such communities are also themselves networks, albeit of a very different type than the networks of data they might create and sustain. The value of communities that are organized as networks and integrated through generalized exchange are particularly apparent when we turn our attention to research on innovation and the public value of science and engineering research.

## 4. The Illustrative Case of Innovation Research

Innovation is critical for improving living standards, advancing health, and self-actualization. The study of innovation allows scholars and practitioners to explore the depth of value created by new discoveries and inventions, including dramatic advances in fundamental knowledge in multiple fields, relevant, practical contributions to some of today’s most pressing policy issues and new technologies. Despite its importance, the innovative process is surprisingly poorly understood.

To see the limits to understanding innovation without linked data and to better understand the value of linked data for studying innovation, it is useful to have a question in mind. Consider a relatively simple example: what is the return on federal investments in academic research and development (R&D)? To dramatically oversimplify things, billions of dollars of grants are made every year by more than a dozen federal agencies to thousands of universities where they support innovative research conducted by hundreds of thousands of people who organize their work in a wide variety of ways.

**Figure 2.**
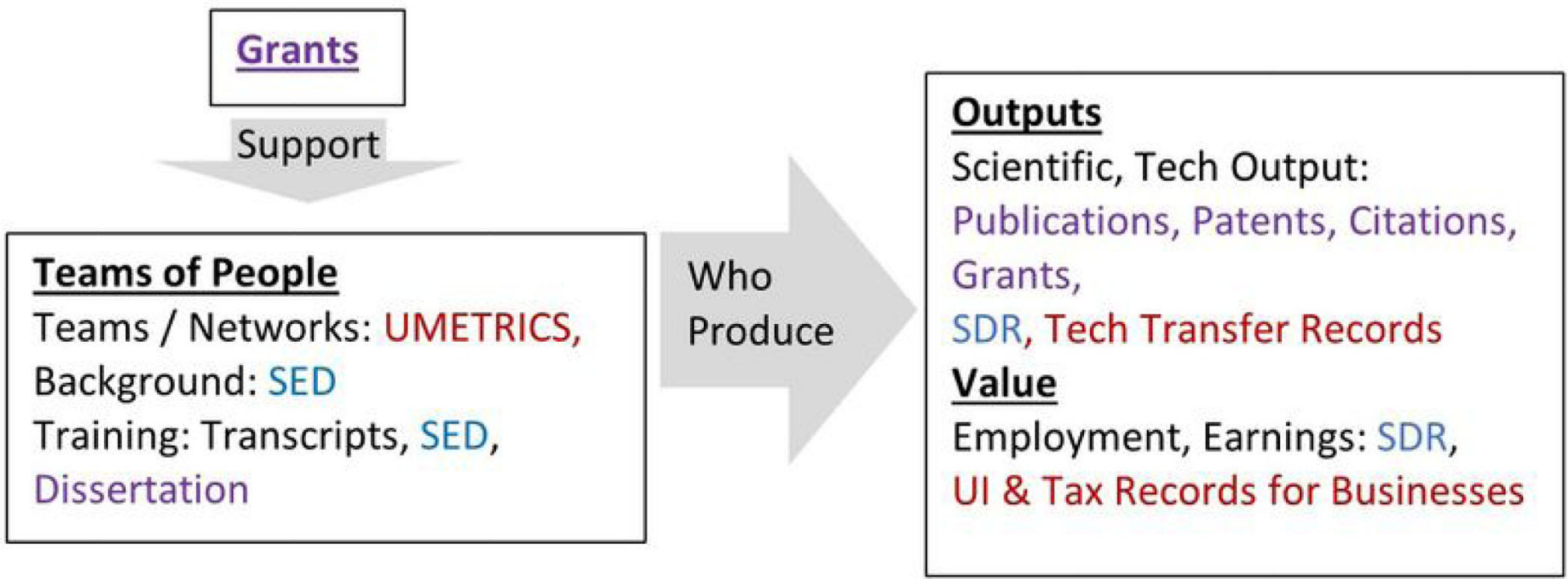
Types of data and roles. The figure illustrates the types of data elements that can be brought to bear to understand how research support affects researchers and teams, output, and the economy. Blue indicates survey data. Red indicates transactional data. Purple indicates naturally occurring data and bibliometric data, which are both unobtrusive. SDR = Survey of Doctorate Recipients; SED = Survey of Earned Doctorates. UMETRICS = Universities Measuring the EffecTs of Research on Competitiveness, Innovation and Science.

However, as illustrated in Figure 2, grants, by themselves, do not do anything. They enable research conducted by people. And, as Corrado and Lane (2009) observe, innovations are made by relatively small numbers of people, frequently working on teams (Wuchty et al., 2007). Their work produces new knowledge while training people to the levels of expertise necessary to effectively apply it. Ultimately, most of those highly skilled people leave the academy for employment across all sectors, where their work impacts the economy and society. Yet, they carry with them the knowledge and skills they gained during their grant-funded work (Stokes, 1997; Zolas et al., 2015). While much of that knowledge is codified in print (Stephan, 1996) or concretized in technology (e.g., patents) or both (Murray & Stern, 2007), much more remains tacit, embodied in the people who produced it (Polanyi, 1966). Private sector access to tacit knowledge largely comes through employment as companies learn by hiring (Palomeras & Melero, 2010; Song et al., 2003).

At a bare minimum, making a start on understanding who research touches and how it affects them and through them society at large requires that we know what specific grants have been made, in what amounts, to what institutions for what kinds of work. We also need to know how they were spent, what inputs were purchased and, critically, which particular people were employed and trained in what kinds of teams. Then we need to know what those teams produced, what role specific researchers played in that production, where those people eventually found other work, what they earned for themselves and what value they produced for their employers. The list could go on and on, but this should be sufficient to make the point—answering even a relatively simple question in this domain requires many types of data (grant obligations and details, granular research expenditures, publication and patent data, demographics, dissertation information, transcripts, employment and wages, and detailed business data to name just a few). These data are housed in many different locations (federal science agencies, university administrative systems, publishers and repositories, federal statistical agencies, state workforce and higher education agencies, firms). Moreover, each of these entities operates under many different sets of restrictions (federal and state laws, ethical protections, proprietary limits on use). Because the focus of this analysis is largely on people, it is inherently sensitive at some level and requires social capital, protocols, and data systems that all of the fiduciaries holding data can rely upon.

Trust in common protocols and systems is essential, but the value of linked data assets can only be fully realized if their use addresses the needs and concerns of all participants. In an area such as ours, where the results of cutting-edge data science can make immediate contributions to both the frontiers of academic knowledge and directly inform pressing policy and social debates, researchers, data custodians, and communicators who make use of results must be engaged at all phases of the work, and all must find value in participation on their own terms. And, for questions revolving around the diversity of the STEM workforce and the breadth of its impacts, it is essential that a diverse community be engaged in data construction and in an analysis.

*Transparency* is essential to both engaging communities and establishing trust in what they produce. This theme is apparent in our work at multiple scales. In technical work with complex data, transparency resolves to clear, complete, and discoverable documentation and the broadest responsible access possible. In the soci-oorganizational work of community building, transparency requires clear processes and practices to ensure equitable and inclusive access to data, to address emergent needs and concerns, and to ensure well-understood and effective protections that meet all necessary legal and ethical requirements while addressing varied participants’ different degrees of willingness to, for instance, tradeoff privacy protections and utility. Transparency also resolves to explicit and sustained efforts to match technical and methodological choices to substantive domain knowledge to help ensure the face validity of work that results.

Finally, *rigor* in the construction, analysis, and use of data and findings is paramount. We take this to mean more than simple replicability (though that too is important). Large, complicated data sets pose challenges in part because they enable a huge range of possible analyses and increase the chance of misuse at the same time. The analytic decisions necessary to render them tractable for particular uses and sensible to relevant communities requires attention not just as to clarity and transparency in technical and methodological work, but also in terms of guiding theoretical principles and concern with the substantive particularities of relevant domains. Large-scale data, we contend, underdetermine knowledge and thus making effective transparent use of them to ensure value for all relevant stakeholders requires that we reach beyond the records and fields themselves in our efforts to render them both sensible and useful.

**Figure 4.**
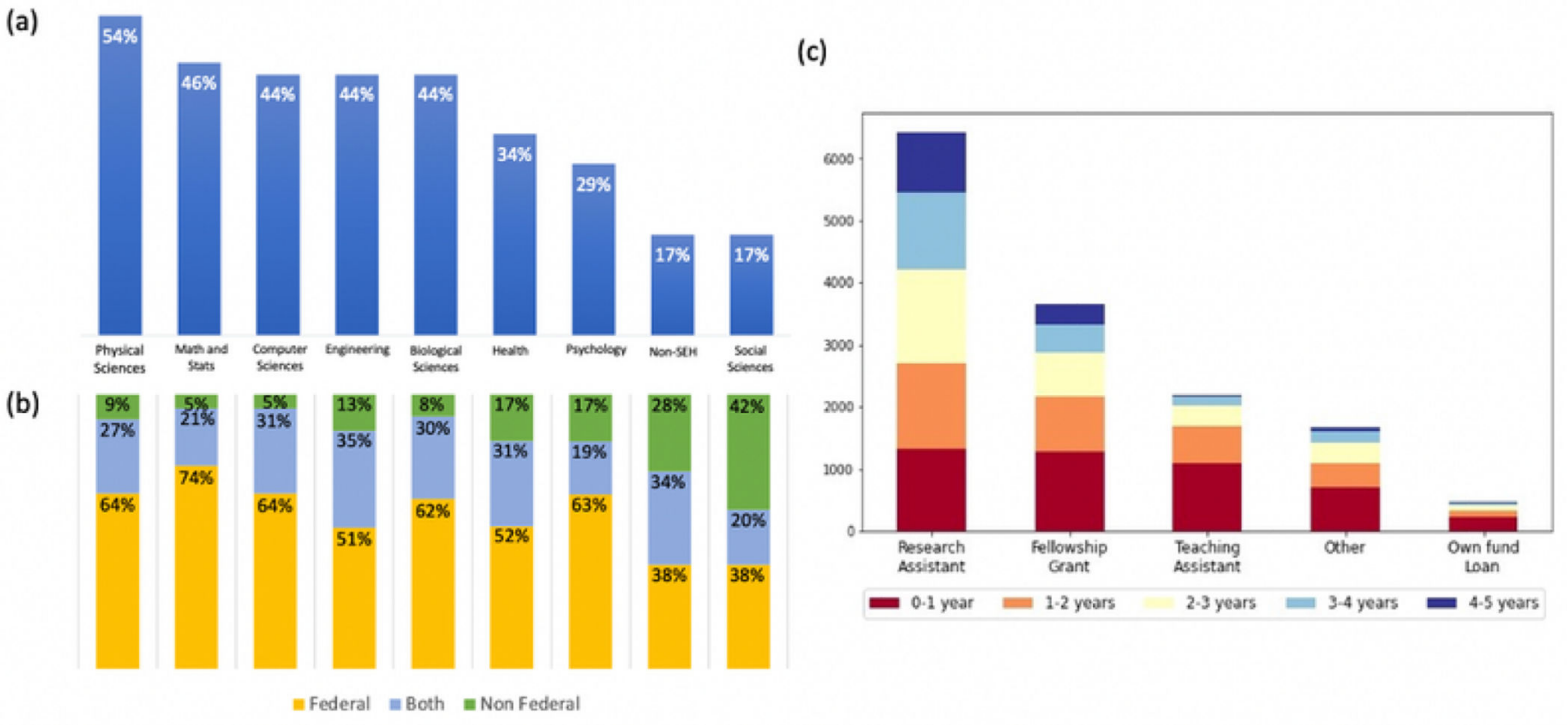
SED-UMETRICS linked data for graduate cohort FY 2014–2017. (a) Match rate by doctoral field of study. (b) Distribution (percent) of funding type by field of study. Funding type is classified into federal source only, nonfederal source only, and funded by both federal and nonfederal sources. (c) Distribution (count) of funding duration by the primary source of financial support during graduate school reported in SED. SED = Survey of Earned Doctorates.

**Figure 5.**
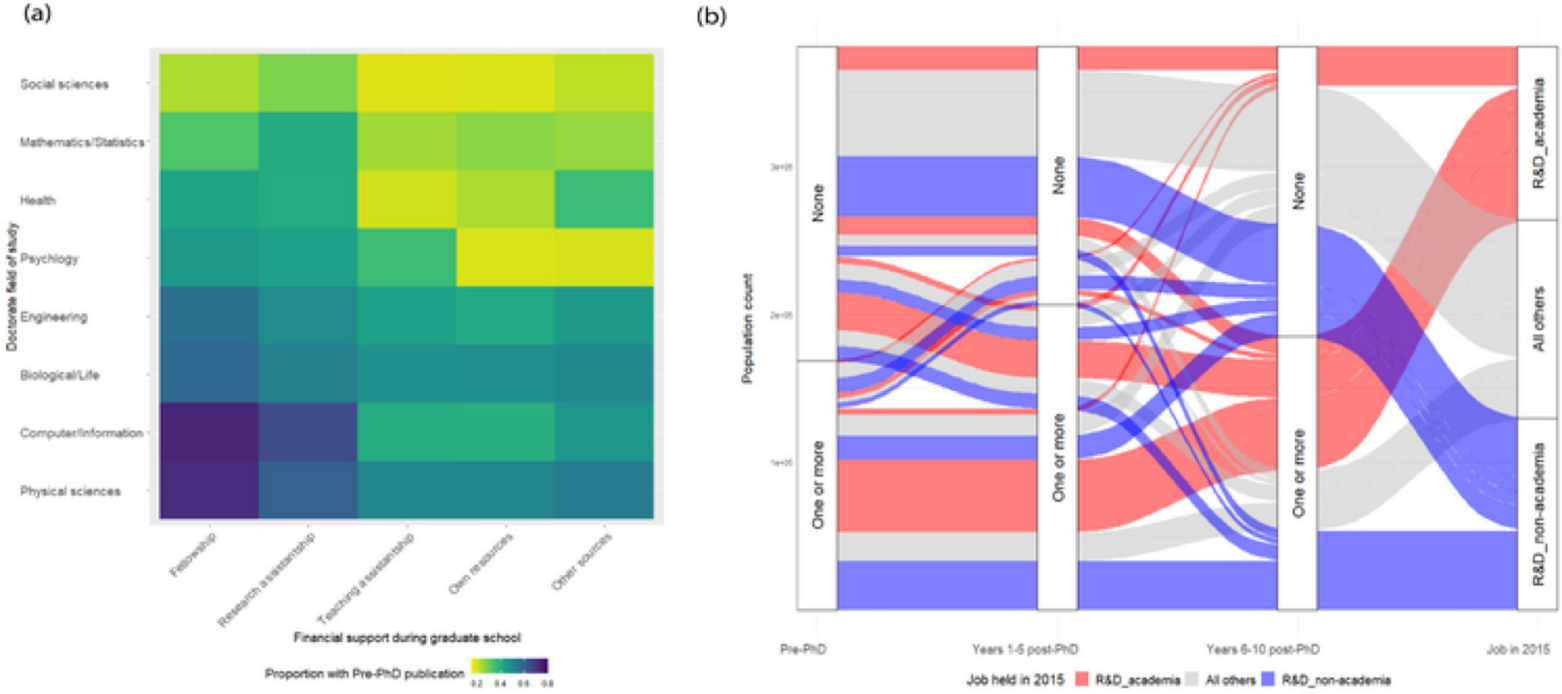
Early career scholarly publishing and employment outcome of the science and engineering doctorate recipients, graduate cohort 1994–2007. (a) Proportion having at least one published article, review, or conference proceeding paper during the pre-PhD period by doctoral field of study and the primary source of financial support during graduate school. (b) Publishing and research trajectories of early career graduates denoted in order of time by indicators of predoctorate publishing, postdoctorate publishing in years 1–5 and 6–10, and employment type in 2015. The pre-PhD period includes 4 years leading to the degree award and the degree year. The 2015 employment type is defined by whether it is an academic position and if performing R&D for primary or secondary work activities.

Speaking to these core principles in a particular area, science and innovation, highlights the general challenges and opportunities of complex linked data in many others, and thus provides broader insights. But all the benefits that might be realized from such work begin with the value that participants derive from it. In the absence of that, the necessary precondition of any such project—data from multiple sources that can be linked—are often impossible to access. In what follows we describe one, concrete, collaborative relationship to build out a single piece of the large data mosaic we envision. The partnership between NCSES and IRIS and the new linked data that results from it (in Figures 4 and 5) offers a case in point.

## 5. Network Example

Ongoing work in the study of science and innovation offers concrete examples of successful network anchors. This article examines two differently organized illustrations and then considers the ways in which their collaboration around a particular data integration task provides concrete insight into both the challenges and the benefits of the data mosaic framework we propose.

### 5.1. IRIS

IRIS is an IRB-approved data repository housed at the University of Michigan, which anchors a consortium of major research universities and maintains productive partnerships with federal statistical and science agencies, foundations, higher education advocacy groups, and other stakeholders in or adjacent to the science policy domain. University members of the IRIS network share transaction-level administrative data on the direct cost expenditures of federal and nonfederal sponsored project grants. IRIS uses these data along with information derived from more than 50 other sources to construct and document an integrated research data release, the UMETRICS data set, that then can serve as the basis for future projects such as those documented earlier. For now, what is important to know is that university administrative data form the kernel of a growing data mosaic that, because of the central role of academic research in the development and value of science, can be extended through responsible linkage to include many types of information at a high degree of granularity. IRIS is a network form of organization and UMETRICS is a network data structure that together represent a growing data mosaic *and* illustrate the conditions for its expansion through partnership with other network anchors that allow linkage to other high-quality data sets.

IRIS uses UMETRICS data for several purposes under protections established in MOUs and other agreements executed with university members and diverse other partners. Three of those uses are particularly important background for the examples and discussion to follow. First, IRIS makes research data, documentation, and support available to a growing interdisciplinary research community of more than 350 people from nearly 150 institutions through a secure virtual data enclave maintained at Michigan, through the Federal Research Data Center (FSRDC) system and, soon, through the Coleridge Initiative’s Administrative Data Research facility (ADRF). Developing and supporting a vibrant research community represents one use for the data the IRIS network produces and protects.

Part of what makes IRIS a network anchor, has to do with the very terms of the memoranda of understanding (MOUs) that support its work. At base, IRIS is designed to absorb two kinds of costs that often impede the collaborative creation of public goods. First, technically, research staff at IRIS collaborate with partners ranging from universities and statistical agencies to individual research groups to clean, improve, and document research data releases. This ‘build once-use many times’ framework results in a common data resource that can be used and expanded for many purposes by many different constituents. Rather than offering partners access to a complete, conclusive data resource, UMETRICS represents a central tile that can be attached to many different data sources to meet the needs of different users. More important than the technical aspects of this work are the institutional dimensions. IRIS is organized to absorb transaction costs that might make more bilateral, decentralized data-linkage projects prohibitively difficult. For instance, research users of UMETRICS data and approved partner organizations, such as the U.S. Census Bureau or NCSES, are able to sign a single agreement with IRIS to access data from many participating institutions. By combining technical and institutional procedures to streamline later data integration and use, IRIS facilitates connections and serves as a network anchor.

Second, IRIS uses UMETRICS, the results of its own and community research, and the skills it has built in working with the data to design, develop, and share valuable data products with participating universities. IRIS routinely produces nine different interactive data products for a variety of use cases—ranging from government relations and communication to faculty and research development and the improvement of education. Universities thus receive direct and tangible benefits from their involvement that would be very difficult to realize on their own. The IRIS community continually updates data and pilots new products in collaboration as research findings create new knowledge and as participants’ needs shift. For instance, rapid turnaround reporting and benchmarking on the research effects of COVID-19 aided many of IRIS member institutions and partner organizations in tracking and responding to the effects of pandemic shutdowns.

Third, IRIS uses data aggregated across all participating campuses, which currently represent about 41% of total academic R&D spending in the nation, to develop and produce public-facing data products used by policymakers, advocacy groups, science agencies, and other stakeholders to forward their own goals and missions in science policy, science communication, and federal and state outreach. IRIS is increasingly turning these privacy-disclosed aggregate products toward communicating the value of science to various publics.

#### Realizing the Benefits.

At its base, then, IRIS is a network anchor that makes transparent, rigorous, and secure use of a wide variety of open access and restricted data from many sources to support fundamental and policy-relevant research and provide valuable benefits to all members of its community. The technical and institutional features that help make it a network anchor by absorbing costs that would otherwise fall to researchers or data custodians and the strong focus on ensuring that streamlined access is accompanied by direct benefits makes the kinds of collaborative integration and improvement work we describe possible. Consider a simple example that will become important in our discussion of linking Survey of Earned Doctorates (SED) and UMETRICS data.

Universities submit monthly transactional data to IRIS. Those data include information on payments of wages from individual grants to particular people. This ‘employee transaction’ table includes information on the job title for which a person is paid on a particular grant and a university-submitted classification of more general occupations (e.g., faculty, students, staff) (IRIS, 2020). Those occupation categories, however, are often incomplete or missing, so university-submitted classifications cannot be used alone for most purposes. Instead, IRIS has, over the course of years, worked with various partners to develop and expand classification systems that work for both research and reporting purposes (Ikudo et al., 2020). This system emerged from work done by university data representatives under the STARMETRICS program, and it was improved by IRIS cofounders Bruce Weinberg and Julia Lane and their research teams while UMETRICS was being piloted with a set of universities from the Big 10 Academic Alliance.

Over multiple iterations of IRIS’s research data release, that classification system has been adapted and improved. Universities receive classifications for their employees with IRIS-produced reports and IRIS technical staff have created mechanisms for university partners to improve the classifications, which they have an incentive to do as a means to make the reports they receive more valuable and usable to local stakeholders. Research teams who access IRIS data for their projects also create improvements using particular methods for specific purposes that can then be tested and where appropriate integrated with future data releases. We shall see that linkage between UMETRICS and SED offers another example, where IRIS classifications of graduate students (based on job titles) can be validated against a universe of known PhD recipients. The result of all this is that IRIS serves simultaneously as a responsible curator for restricted data for many purposes and as a location where different parts of a larger community can collaborate to improve a shared resource. This is the context in which we work to mitigate the dangers of opportunism and competency shortfalls.

#### Addressing the Dangers.

Minimizing opportunism requires formal and informal arrangements designed to promote fairness and transparency in procedures while also supporting inclusiveness and accessibility in operations and data access. IRIS strives to accomplish this by making all requirements and materials for data access public along with easily accessible and clear documentation, ready and equitably distributed administrative and research support, and encouragement for data and expertise sharing within groups (e.g., researchers and data producers) and across them.

A key part of this work is ensuring not only that access and support are transparent and fair but also that there are mechanisms to allow all members of the community to receive credit for work they do that contributes to the common good. With credit comes credibility for IRIS and, more importantly, a reward for individual community members engaged in the work of generalized exchange. A major source of value for research community members is recognition for completing quality work that is documented and freely shared. One means to limit opportunism is to facilitate mechanisms for data users and data providers to establish positive (or negative) reputations for themselves, which can help guide the collaboration decisions of their future partners. Both of these mechanisms benefit from transparency and reinforce procedural fairness. They are augmented by mechanisms such as research support for documenting and archiving code and measures as well as replication data sets. Collaborative tools (such as secure git repositories within the IRIS virtual data enclave) and means to discover, disseminate, and cite user-generated work (such as IRIS-minted DOIs) aid in both these processes while helping to make certain that the network IRIS anchors do not become closed to new ideas, new techniques, or new analysts.

Running throughout the whole of our argument is the theme of value for participants. Given that we cannot and should not rely on altruism or preexisting bonds of affinity or kinship (which could themselves contribute to network closure and calcification), the pursuit of individual interests and goals that can only be met with inputs from others becomes a primary driver of participation and spur toward generalized exchange. IRIS directly ensures value to data providers, which builds trust that facilitates the creation of streamlined data protections that, in turn, put access to data within reach of a wider community and increase the range of types and sources of data that are available. As the quality, scale, and scope of data resources increase, so too does the value for all members of the community. Perhaps unsurprisingly, shared, community-driven data resources and vibrant generalized exchange networks have positive externalities, reinforcing a virtuous cycle of credit and engagement while further diminishing the likelihood of opportunism by increasing the amount one stands to lose when bad behavior is identified.

Taking advantage of the many sources of value that are ‘baked into’ joining and fully participating in the IRIS network also requires attention to costs. The fundamental cost of complex collaborations that involve data sharing and integration are as much transactional as they are technical or financial. Indeed, the challenge individual researchers would face in overcoming the frictions necessary to secure data access permissions from a wide range of providers would be insurmountable for most. The work to build trust and establish value that is core to the IRIS model allow it (with the help of well-crafted agreements) to minimize the transaction costs of data access and collaboration for all. IRIS negotiates dozens of agreements with provisions that allow us to make the full data set we produce available to researchers under a single agreement with no additional university approvals.

Likewise, non-university data providers can, with appropriate approvals from IRIS’s elected board of directors, access data and link assets from many sources with a single agreement. Establishing the value of the data set as a whole for aggregate reporting on matters of pressing concern to non-university partners increases the likelihood that future data-sharing agreements will augment value for everyone. The benefits of participation compound to limit the likelihood of opportunistic behavior by lowering the cost of participation through streamlined data access and increasing its value through growing scale and scope. Here too, reciprocity becomes an essential, and positive, force for community development. The linkages we present between SED and restricted transaction data from nearly two dozen different institutions offers just one case in point.

Transparency, fairness, accessibility, value, and efficiency may seem far removed from the day-to-day details of developing a research community. But they are essential accelerants for generalized exchange and retardants for opportunism.

The primary means IRIS uses to decrease the dangers associated with screwing up flow from and reinforce these principles too. If one primary danger for data science communities is competency shortfalls among participants, it becomes essential to meet data users and data producers where they are. Building research community is thus inseparable from building collaborative, technical, and analytic capacity. That capacity, in turn, improves transparency and increases the rigor of both individual and collective work.

Training opportunities, public convenings, and clear documentation and support for mechanisms to allow learning within and across constituent groups increase accessibility and the flow of new people and ideas into the network. They also reduce the forms of ignorance that can lead to unintentional screw-ups. IRIS routinely offers a 9-day-long virtual workshop series aimed at providing novice research users of largescale restricted data with the tools they need to get started. Sustained collaborative work and substantial technical support in those settings jumpstarts expertise building and generalized exchange. Other courses, which IRIS contributes to, are run by our partners NCSES and the Coleridge Initiative. Those courses aim to engage analysts and technical staff at federal and state agencies as well as universities and they rely directly on the analytic data sets whose production we describe in some detail while providing more description of the classes themselves in a subsequent section. Training opportunities such as these serve not only to increase capacities for collaboration but also to further integrate disparate but interdependent portions of the community and to help members with different backgrounds and expertise learn from and communicate with each other. The macroscopic results of all that work refresh the network, expand the grounds for generalized exchange, and decrease the likelihood of both screwups and screwings.

### 5.2. NCSES

NCSES is a leading provider of statistical data on the U.S. science and engineering enterprise. As a principal federal statistical agency, NCSES serves as a clearinghouse for the collection, acquisition, analysis, reporting, and dissemination of statistical data related to the United States and other nations. NCSES conducts more than a dozen surveys in the areas of education of scientists and engineers, R&D funding and expenditures, science and engineering research facilities, and the science and engineering workforce with type of respondent ranging from individual, academic department and institution, state agency, federal agency, to company, business, and establishment. In recent years, NCSES has explored alternative data sources in conjunction with survey data to bolster the utility of its surveys and avoid imposing further response burden on the survey participants. NCSES also supported requests to match its restricted-use survey data with administrative or other data as potential research tools.

Results of these data linkage explorations formed a basis for a research data network centered around the SED, an annual census of individuals who earned research doctoral degrees from accredited U.S. academic institutions. These census data of the highly trained and highly invested U.S. research doctorate holders is a natural magnet attracting data-matching requests to study outcomes of federal programs such as those supporting diversity in doctoral training (McNaire Scholars program), engineering workforce development (National Science Foundation [NSF] Engineering Research Centers), and research opportunities for undergraduate students (NSF Research Experiences for Undergraduates program). For this population, postgraduation career trajectory, scientific productivity, and their contributions to innovation are also high-priority topics in science policy research. Connected to the SED by design is a NCSES workforce survey, the Survey of Doctorate Recipients (SDR) a biennial longitudinal survey. The two linked surveys provide rich demographic, education, and career history information for this population, and significantly increased feasibility of further expansions through data linkage to illuminate policy-relevant topics ranging from the impact federal research funding has on graduate education, job placement, career progression, and research output as well as scientific mobility indicators, factors impacting scientific workforce diversity, and gender gaps in publication productivity, earnings, and employment (Goulden et al., 2011; Kahn & Ginther, 2017; Millar, 2013).

Data linkage involving restricted-use data is governed by the center’s matching policy, which requires work to be performed by NCSES contractors or, with NCSES approval, by a federal agency. The center’s practices in protection of the confidentiality of NCSES collected data and statistical standards placed on data production add cost and operational challenges on top of the technical challenges of developing linking methods. For example, contractors are required to have a NCSES-approved confidentiality plan and data security procedures before the work can begin and it historically entails building a customized secure data system for the project. At times, this practice and data use agreement can take a significant amount of time to establish. Not until 2019, when NCSES started housing-linkage projects in the NCSES Secure Data Access Facility (SDAF), did linkage projects begin to flow more efficiently. Using the same facility, where NCSES also provides secure remote access to confidential survey data for licensed researchers, cross-pollination between project teams and collaborations with data users that have shared data interests could be efficiently facilitated and managed effectively by NCSES.

Many questions of interest for the population of doctorate recipients revolve around research publications, and the SDR has periodically included self-reported data on publications. At the same time, a wealth of data on publications is available in publication and citation databases. These data constitute valuable information that, through linkage, might augment the SED/SDR without increasing burden on respondents. NCSES partnered with the research community in multiple ways to conduct this work. First, linking respondents in the SED/SDR to authored scientific publications and U.S. patent inventors is a NCSES major research undertaking that began in 2012. The linkage methodology continues to evolve from an initial deterministic matching attempt to advanced machine learning approaches. All the while, venders of bibliometric data continue to improve data coverage and offer new data features that could redirect the linkage methodology. The first pilot project with Clarivate (then Thomson Reuters) delivered results in 2016. Staff reshuffling during the lengthy project with the departures of key technical personnel and project managers resulted in incomplete linkage quality assessment and process documentation. NCSES engaged the community to complete data-quality assessments and prepare for data release, contracting with three independent research groups. One (Science-Metrix) conducted a review of the potential data source biases and methodology shortfalls, one (Ginther et al., 2020) performed independent validation of matched records of publications, and one (Jiang et al., 2021) performed analysis of matched publications and provided guidance on how to use the linked data. Valuable information was gathered on linkage quality and data limitations. Here NCSES was able to facilitate the construction of a data mosaic, supporting linkages that would require resources beyond those of most or all research groups, address privacy and confidentiality issues, and engage community expertise to assess those linkages to produce high-value data for the community.

**Figure A1.**
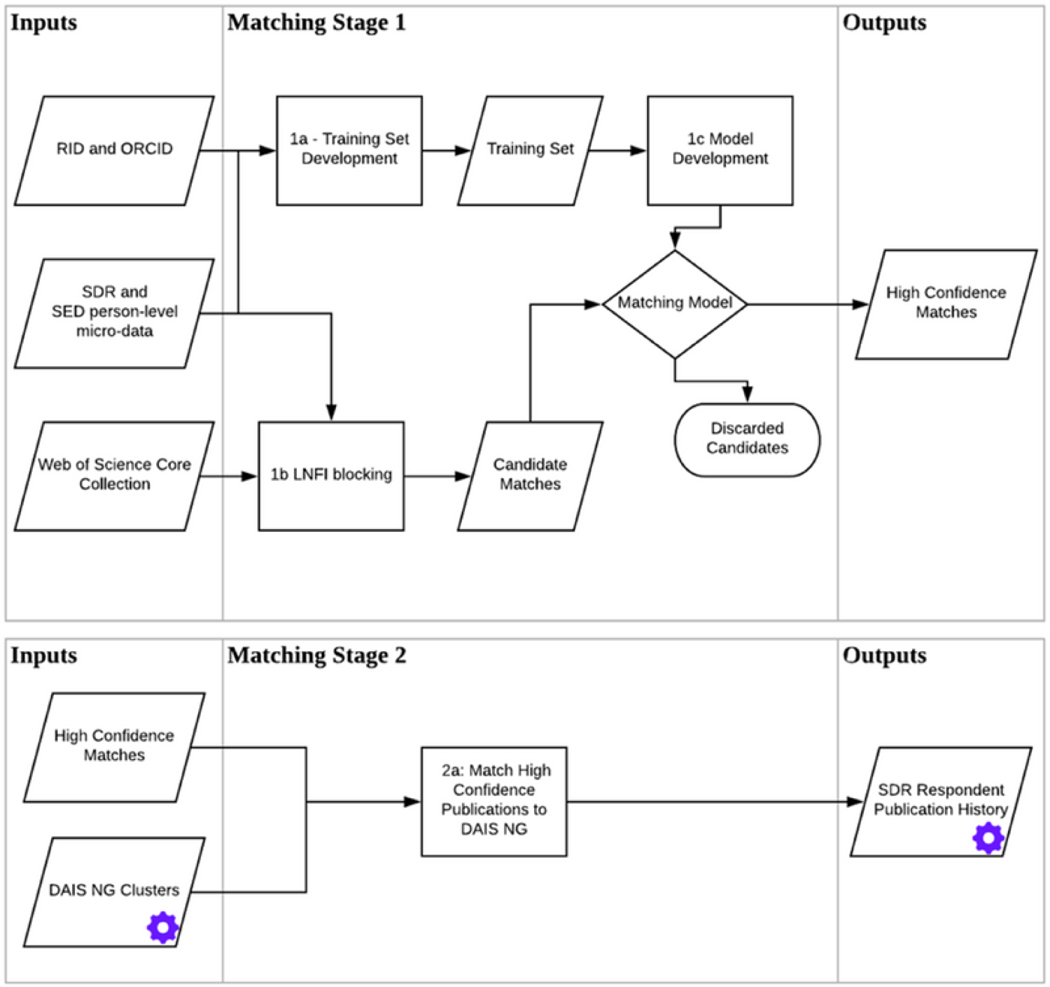
Matching takes place in two stages. Stage 1 produces high-confidence matches for the Survey of Doctorate Recipients (SDR) respondents and Stage 2 expands the matching process by utilizing WoS Author Clusters (DAIS NG). The gold standard data, manually curated publication histories developed for two sets of SDR respondents each, 800 REFID and 785 REFID, were used in two places during this process as indicated by the purple gear icons: (1) a training set and (2) a test set used to conduct postprocessing targeting 95% Precision during Stage 1 and to produce a final assessment of precision and recall after Stage 2. LNFI = Last name plus First initial; REFID is the SDR person identifier; SED = Survey of Earned Doctorates.

NCSES kicked off a new round of linkage in 2019 to link a new and larger sample (~80,000) from SDR 2015 to the two leading bibliometric databases, Scopus and Web of Science (WoS). By contracting with both Elsevier and Clarivate, NCSES plans to explore contractor capability and identify a longer-term partner to build a repeatable and sustainable data production. The Elsevier team utilized a well-established author ID system and a sophisticated query algorithm to link SDR respondents directly to Scopus author IDs using all identifiers and linkable attributes from the cluster of publications associated with each author ID. The Clarivate team took a sequential two-stage machine learning approach as detailed and illustrated in Appendix Figure A1. In stage 1, high-confidence matches were determined by applying a supervised gradient boosting machine learning algorithm (GBM) constructed with 67 features ranging from comparable direct and indirect identifiers to auxiliary variables containing only information from the SDR (e.g., race/ethnicity) or Web of Science (WoS) (e.g., name commonality.) In stage 2, the resulting high-confidence publications were used to seed into and selectively append additional publications from WoS Author Clusters, an independent methodology that disambiguates the more than 171 million WoS publication records.

Lessons learned from initial pilot work made it clear that (i) model performance is not uniform across subgroups and prediction thresholds must be adjusted to reflect limitation of source data to achieve the required high-precision level (for example, individual author affiliation data are not available for all years or all types of publications); (ii) linkage errors need to be assessed at levels aligned with the expected unit of analysis (in this case, at the individual publication and individual researcher levels); (iii) most of readily available bibliometric measures are derived for single publications, and meaningful measures of publication output and impact are needed at the researcher level to support population estimation and reporting. This activity relies heavily on analysts from both bibliometric and survey sides to collaborate; and (iv) a new set of statistical standards need to be developed to support releasing of research microdata by a statistical agency.

### 5.3. The IRIS-Coleridge-NCSES Partnership

If the UMETRICS and NCSES data architectures are both evolving mosaics, the next step is to combine these mosaics. The exponential value of data mosaics suggested in our toy model is a function of just this kind of extension, where a strategic linkage (in this case between UMETRICS and SED) conducted through an ongoing collaboration opens a wide range of new possibilities for improving access to and use of both the newly connected mosaics. This work is in progress under a NCSES contract with the Coleridge Initiative and being conducted in the Administrative Data Research Facility (ADRF). Combining these data sets adds considerable value to both, as is illustrated following. For instance, the SED is administered at a single point in time, although it has retrospective questions. Adding UMETRICS adds dynamic richness. For UMETRICS, which does not contain data on field of study or background, the SED provides context. Of course, UMETRICS also provides context to the SED in that the SED contains limited information on researchers’ formative training experiences in the form of funding and research teams, while UMETRICS contains data on the source of research support (funder and specific award) as well as the research teams in which people are embedded.

The key to realizing the value of this linkage beyond the specific projects come from: (a) the process by which the linkage process draws on complementary expertise of network anchors, (b) the enabling features of the data platform (ADRF), and (c) the institutional arrangements of IRIS that allow for integration of data from many institutions and sources with NCSES surveys under conditions that minimize transaction costs for NCSES but that maintain necessary data protections required by both partners.

All of these features are enabled by both parties’ recognition of and attention to multiple sources of value. NCSES and IRIS share the goal of making well-documented, linked data that can address key questions in the study of science and innovation broadly and make it responsibly available to a diverse research community. The partnership thus reflects a common recognition of just the kind of value our model abstractly identifies with linked data mosaics. This common source of value is matched by independent needs for each organization, which the joint project helps to address. For IRIS and its member institutions, linking university administrative data to a widely used and recognized ‘gold standard’ data set for understanding graduate education represents the potential for important new findings and data products that might be developed to address key concerns of universities and their graduate programs that pertain to diversity, equity, and inclusion and to widespread efforts to re-envision STEM graduate education for the 21st century (NASEM, 2018a). Thus, the partnership has immense potential value to IRIS and its stakeholders.

For NCSES, streamlined access to administrative data from many institutions and collaborative means to develop expertise in their use likewise address specific needs. Recall that, in its role as a network anchor, IRIS is designed to absorb transaction costs and to facilitate the development of community expertise in the use of the data it creates. Because of these features, NCSES can sign one agreement to access many institutions’ data and the collaborative work to develop linkages (discussed following) can grow each organization’s relevant expertise. This work thus addresses both requirements under the Evidence Based Policy Act and Federal Data Strategy and recent recommendations made by a NASEM panel that evaluated NCSES data products (NASEM, 2018b). Training classes that use the linked data this partnership produces serve both organization’s needs and their joint goals by developing local and community capacity and deeply engaging researchers in the process of expanding and documenting data linkage.

### 5.4. Technical issues in Linking SED and UMETRICS

Realizing the value of this partnership has required consistent, long-term, and iterative work that engages analysts from IRIS, NCSES, and the Coleridge Initiative as well as participants in four training classes. While our goal is not to dwell on the deep technical details of this particular linkage, which largely rely on standard methods in data science, we do highlight key questions and processes that we take to be necessary for the project’s success. We summarize them here in the belief that similar issues are likely to arise in data integration efforts in this and other fields and hope that the discussion will illustrate common critical challenges and illuminate in very concrete terms aspects of the data mosaic approach we propose.

#### Units of Analysis.

Earlier, we noted that one of the first substantive questions for any data mosaic project has to do with establishing a small number of fundamental levels of analysis for the overall effort. In this pilot effort, as in the larger initiatives of which it partakes, that level of analysis is the individual researcher, or, more accurately, the individual research career. This decision was motivated by the exemplary discussion in section 4 and represents a key distinction between this data linkage effort and other large-scale projects relevant to our domain, which often focus specifically on documents, publications, patents, proposals, and grants.

#### Linking Variables.

The selection of that level of analysis is driven by theoretical concerns, but also by substantive knowledge of the field and by the needs of participating community members. It is important for the general project of data linkage because the fundamental level of analysis dictates the key linking assets and also the levels and types of privacy protections required. In our case, we rely on personal identifiable information (PII), either hashed (in the case of SED-UMETRICS linkage) or clear text (in the case of SDR-WoS) linkages. The SED-UMETRICS linkage is augmented by information on each individual research employee’s month and year of birth. Those fields, rather than complete date of birth information, were selected in concert with university data providers after careful pilot tests of linking procedures that helped us determine the highest value assets for improving linkage. While adding day of birth to these data would offer a minor improvement in linkage quality, that improvement came at the cost of expanded concerns about privacy and risk and thus we chose not to pursue the additional data asset. Such risk-utility tradeoffs with regard to privacy and security are best driven transparently by discussion of community needs and value, and represent another general challenge for this type of project.

#### Protecting Privacy.

A fundamental concern for linked individual data is the increased danger of primary and secondary reidentification that accompanies a dramatic increase in information associated with individual-level identifiers. This danger is particularly salient in fields such as health care and education (which this project implicates) where federal regulations such as Health Insurance Portability and Accountability Act (HIPAA), Federal Education Rights and Privacy Act (FERPA), and Confidential Information Protection and Statistical Efficiency Act (CIPSEA) impose specific requirements on participants and on research users. A substantial challenge for bringing together data from two different organizations with their own privacy and security protocols is finding and agreeing upon an environment that will allow the members of the prospective organizations to collaborate while upholding their separate privacy and protection requirements.

IRIS and NCSES agreed to have the SED-UMETRICS linkage conducted within the Coleridge Initiative’s ADRF. The ADRF is a FedRAMP Certified secure cloud-based computing platform designed to promote collaboration, facilitate documentation, and provide information about data use to the agencies that own the data. The U.S. Census Bureau established the ADRF with funding from the Office of Management and Budget to inform the decision-making of the Commission on Evidence-Based Policy. The ADRF was designed to apply the “five safes” framework (safe projects, safe people, safe settings, safe data, safe exports) for data protection (Desai et al., 2016; Arbuckle & Ritchie, 2019). Since the ADRF was designed to bring together data from different organizations and facilitate collaboration, it provided the framework to establish data-security protocols that met the needs of both NCSES and IRIS. Still, developing a protocol to establish safe people and safe exports was not a trivial process.

We match individuals in the SED and UMETRICS data sets on name, institution, and date of birth information. To meet IRIS’s privacy and protection requirements, the personally identifiable information (PII) had to be hashed before bringing the data together in the ADRF. To realize this requirement, we apply the same standardization techniques to information from each data set and hash the resulting strings using the same algorithm and seed. The result is that identical cleartext PII strings are transformed into unique, identical hashed strings, which can then be used for deidentified linkage to integrate the two data sources. Since the personally identifiable information is hashed, our options for linkage are restricted to using an exact matching strategy. We are unable to use a sound-matching or statistical similarity approach.

This exact matching strategy across multiple name-token combinations gives us more confidence in the matches we identify; however, the relative inflexibility of our approach will fail to link individuals who appear in both data sources with a misspelled first or last name in one, and it will also face challenges where survey data contains nicknames or other substantive differences between an individual’s self-identification and their formal legal name as registered in university administrative systems. Parallel data-cleaning processes applied to both data sources included a step that harmonizes full names and common nicknames associated with them. Nevertheless, the tradeoffs involved in these privacy protection procedures may lead us to underestimate the number of individuals who receive grant funding while earning a PhD. Plans to conduct parallel cleartext PII linkage of UMETRICS and SED once appropriate institutional clearances are secured will allow for additional validation. But, in keeping with the mantra of establishing value while hewing closely to the privacy requirements of particular communities, an imperfect initial match can be used to demonstrate value and build further support for expanded work with these restricted data.

Similarly, the SDR and WoS linkage was carried out within a closed, secure data-access environment, NCSES’s SDAF, to safeguard the privacy and confidentiality of the survey respondents. The constraints added to the operational cost and efficiency. The data construction does not end at the completion of data linkage. In the case of appending longitudinal publication outcomes to surveys rich in demographic information at the individual level, the risk of reidentification can increase dramatically. We face new challenges in designing research data that can be disseminated broadly to the communities while addressing the heightened disclosure risk. Here, too, the dictates of data structures and privacy requirements are a key component of analytic decision-making in early project stages.

#### Identifying a Cohort With Nonoverlapping Samples.

One of the first issues that arises in any linkage, which confronted the work we describe, is the timeframe to use. The SED has covered all accredited U.S. institutions since 1957; therefore, its duration and timeframe was not a limiting factor when considering the appropriate timeframe for analysis. On the other hand, the availability of UMETRICS data varies across universities and ranges from 3 months to 18 years, with an average of 7 years and 8 months. The earliest UMETRICS data available begins in 2001; however, the number of reporting universities is sparse in the earlier years, with fewer than half of the universities available before 2013 (IRIS, 2020). We have the most coverage for the fiscal years 2014–2017. Each of the 21 universities we analyze here is represented during this time; 16 universities have full coverage, and the other five universities are only missing one year. Thus, we focus on PhD graduates from SED during the fiscal years 2014–2017. (One of the IRIS universities only had 3 months of UMETRICS data available for this time and thus only captured a fraction of the grant funding from the doctoral graduates; thus, we removed this institution from the analysis.) As with any such decision both data sufficiency and technical needs must be balanced with substantive concerns (e.g., changes over time in fields).

Another, potentially related, issue that can arise is how to harmonize time across data sets. This was less of an issue in our context because the SED is administered at a single point in time while UMETRICS is longitudinal. On the other hand, there was more of an issue to develop standardized time variables within UMETRICS. While it would be possible to provide full-time detail, there is an advantage to providing a more parsimonious set of variables. For many purposes, we chose to provide data at a monthly level, which still provides considerable richness, but with more parsimony.

#### Validation Strategies.

When producing a linked data asset, conducting the linkage is only the first step of the process. After the linkage is performed, steps must be taken to ensure the integrity and reliability. In our case, this process was a collaborative effort between IRIS, NCSES, and the Coleridge Initiative for the SED-UMETRICS links and between NCSES and Clarivate for the SDR-WoS linkages. It was only possible due to the deep understanding of the separate data sources that each organization brought to the table, which was crucial for identifying the type of validation processes that were possible and necessary to produce a responsible, reliable, and secure combined data asset. Here, too, both broad domain knowledge and the benefits of combining deep sources of data set–specific expertise underpin the potential value of the linked data that result. Instead of giving an exhaustive list of the validation checks used, we will highlight a few, while focusing more on why they were conducted and how data analysis, owners, and communicators might think through similar processes when constructing future linked-data assets.

When a linkage is conducted through many steps with less strict criteria at each step, as was the case for our SED-UMETRICS match, it is essential to ensure there is a valid reason that match could not be made during a previous step. When possible, it is also important to quantify the loss of precision that results from the less-restrictive matching criteria. For example, the SED-UMETRICS matching procedure was performed through six steps, with the matching rule relaxed with each step. For matches made during odd steps, the full name had to match, while only the first and last name had to match for matches made during even steps. We sought to understand why matches were made using only the first and last name when the full name did not result in a match during the previous step. We found that most of the time, this was due to the middle name missing in one of the data sets or the middle initial being recorded as the middle name in one of the data sets, thus preventing a full name match. In later steps, we also relaxed the date-of-birth criteria to accommodate missing date of birth information, requiring the date of birth to be absent in at least one of the data sets. This prevented matches from being considered who had a different (valid) date of birth information in the two data sets, but permitted matches where one of the dates of birth was empty. We also quantified the loss of precision associated with the less-restrictive date of birth criterion, estimating a loss of precision of 21% from steps 3–4 and 23% from steps 5–6 compared to steps 1–2. Applying this less-restrictive matching criteria resulted in an estimated 267 false positive matches, while not applying the less-restrictive matching criteria would have resulted in an estimated 894 false negative matches. This process is discussed further in Appendix B.

Running this analysis not only increased our confidence in the matches made during subsequent steps, it also helped us identify three principles that will be useful for future linkages that use hashed PII data. 1) When preparing and hashing data from separate data sources, it is helpful to hash both the a single field with all PII elements and to separately encrypt specific subsets of PII such as first and last name. This allows more flexible implementation of linkage algorithms. As discussed, having the hashed middle initial available allowed us to gain much greater insight into why matches were made during even-match steps instead of the previous odd-match step. 2) When linking on hashed data elements, it is important to be willing and able to think out of the box and use all the information available to gain a deeper understanding of the linked data. If the data were not hashed, it would be straightforward to look at the raw data to identify middle initials recorded as middle names; however, raw hashed data shows a random 64-character string recorded as the middle name in both data sets with no identifiable connection between the two strings. 3) Linkage errors often arise when data-linking efforts are undertaken without direct involvement of the data providers (Gilbert et al., 2018). When linking hashed data, it is impossible to overstate the importance of working with the data providers who have a deep understanding of the different data sources. For example, during our first attempt at linking the data, we followed *Linking the Survey of Earned Doctorates (SED) to Administrative Records* report by the American Institute for Research (2015), and only matched individuals who were classified as graduate students in UMETRICS. However, after discussing the occupation classification process with experts from IRIS, we decided that the noisiness of the occupation classification (Ikudo et al., 2020) resulted in an over-restrictive match set. Thus, we removed the graduate student classification requirement from our matching algorithm.

## 6. Training Programs

Just as supporting and facilitating exchange within the community is key to data mosaics, a key component in linking SED and UMETRICS is engaging the community to pilot the data and guide and inform the linking process. NCSES and IRIS have partnered with the Coleridge Initiative to conduct a series of applied data analytics training programs to engage analysts and technical staff at federal and state agencies and universities. During these training sessions, participants are divided into four to six teams, and each team develops a unique research project using the linked SED-UMETRICS data. This process provides insight into the types of research questions the community is interested in answering with the linked data and informs validation checks performed to test the quality of the match. The process of ensuring that a wide range of community members develop the capacity to effectively use and improve this data mosaic is inextricably tied to its ongoing development and that connection is a key source of vibrancy.

For example, many teams have been interested in gender disparities in federal funding. For this linkage, we have a gender-match rate of 32% for females and 39% for males. The participants also pondered whether the discrepancy in match rates reflects true disparities in funding or if it is driven by matching-bias concerns that stem from the custom of women changing their name after they get married. To distinguish between these hypotheses, during the validation process we tested to see how marriage affected the match-rate discrepancy. We found that there was a greater match-rate disparity for unmarried women than unmarried men when compared to their married counterparts (Chang et al., 2022).

In addition to informing the matching and validation procedures, the training program increases collaboration capacities and further integrates disparate but interdependent portions of the community. For instance, a 2021 class piloted the linked SED-UMETRICS data. NCSES, IRIS, and Coleridge also partnered with Excelencia to provide a training program where all participants were affiliated with HBCUs or HSIs.

During the training program, participants work with people from different agencies and universities, sharing ideas, knowledge, experiences, and code. This experience helps unite and build the community around the linked data. It even helps get new data providers excited about contributing their data to the data mosaic. We have had participants from agencies that award research grants bring in additional data on such grants, with varying degrees of success in linking their data to the SED-UMETRICS data provided during class. The training opportunities thus provide a key opportunity for learning on the part of IRIS and NCSES and an essential source of momentum for continued expansion, accessibility, and use. Perhaps more importantly, routine capacity-building efforts of this sort help to build both the kinds of reciprocal exchange relationships among data providers and users that promote generalized exchange (and limit the likelihood of opportunism) and aid in the creation of community wide norms and practices for responsible, effective data use (limiting the possibility of damaging screw-ups).

## 7. Initial Results

### 7.1. SED-UMETRICS

How do the trajectories of research support experienced by researchers in different fields compare? As discussed in section 4, even such a basic question is not answerable using either UMETRICS or the SED on its own because UMETRICS does not have data on field of study, while the SED contains some information on broad categories of support but does not provide information on the timing or length of support.

We have completed initial matches between the SED and UMETRICS for 12,066 graduate students from 21 UMETRICS institutions who completed their doctorates during academic year 2014–2017. We use this initial match to provide an early glimpse into the potential value of linking these data sets to answer questions about how research support trajectories vary by field.

Recalling that UMETRICS comprises data on people supported on research projects, we proxy for the receipt of federal or nonfederal research support using whether someone who appears in the SED is matched to a UMETRICS record. (We acknowledge that linkage errors constitute another reason why records may fail or be incorrectly matched.) The overall match rate calculated as the proportion of SED doctorate recipients from UMETRICS universities who link to UMETRICS is 36%, suggesting that a sizeable minority of graduates receive any research support. Yet, that overall match rate hides significant variation by doctoral field of study. Social sciences and non-SEH are the two broad fields with the same lowest rate of 17%, while physical sciences has the highest match rate of 54% (Figure 4a). As expected, fields (such as social sciences and non-SEH) that are less reliant on external funding to support doctoral training have substantially lower match rates.

These links also have the potential to shed important light on disparities across groups because data on gender, race, and ethnicity in UMETRICS were produced using imputations based on names, while the SED contains individual self-reports. Marked differences at the intersection of race, gender, and discipline in the granting of doctoral degrees (Chang et al., 2022) are an important factor contributing to the observed differences in match rate by gender and race. For instance, the match rate for African American doctorate recipients is 25% and that is consistent with a majority (51%) receiving their degrees in the two broad fields with the lowest match rates; however, disparities still exist within some broad degree fields (Chang et al., 2022).

While the SED contains information on broad categories of support (e.g., research assistantship, teaching assistantship), it does not contain information on the source of that support (e.g., federal or nonfederal) and it is not clear that individual respondents would be able to accurately state whether the funding for a research assistantship, for instance, was from a federal source. Moreover, the SED does not provide any information on the length of support. UMETRICS allows calculation of both source of research support and the time path of support, but lacks field of degree information. Their combination offers unique insights and novel opportunities.

Focusing on research assistantships, which the SED shows to be the most common form of support for doctorate recipients during graduate school, panels B and C provide deeper insight into doctoral student funding than could be had with either source of data alone. Panel B shows that among those matched to UMETRICS, the overwhelming majority of research support is from federal sources. Moreover, it is informative to document both where fields differ and when they are the same. For instance, 42% of matched social science doctorate recipients rely only on nonfederal funding sources for support, while much smaller percentages of students (5%–17%) in other science and engineering fields rely exclusively on nonfederal support. Roughly half of students in engineering and health fields are supported only on federal research projects, but slightly over a third of students in both fields are supported by federal and nonfederal projects. Both differ significantly from physical sciences, a pattern we found surprising and worthy of further exploration.

Panel C shows how UMETRICS can contribute additional temporal richness to the SED’s wealth of current and retrospective data. For instance, it makes it possible to investigate the how the length of time that people received support on research projects varies by the type of support. Thus, the share of people who receive 0 to 1 year of research support in UMETRICS is more than 1,000 for people who report being supported primarily as research assistants, on fellowships and/or grants, and as teaching assistants, but roughly 5,000 people who were primarily supported as research assistants were supported for 2 to 5 years compared to roughly 2,000 people supported primarily on fellowships and/or grants and roughly 1,000 people supported primarily as teaching assistants.

### 7.2. Initial Results: SDR/SED-WoS

As discussed in section 4, just as it is important to be able to link researchers back to the sources that supported them, it is valuable to be able to link them to their research outputs and career trajectories. Currently, the SDR, which is drawn from the SED, has been linked to the Web of Science, which includes 1,220,565 publications and 1,344,260 SDR respondent-authorships pairs, disambiguated from among more than 160 million pairs, for 57,874 (73.89%) doctorate recipients who responded to the 2015 SDR.

The SDR/SED-WoS linkage provides data to align the publishing timeline with doctorate degree award time and other career milestones and allows us to relate publication trajectories to source of support as well as field of degree. The rate of scientific publishing is known to vary by field (Larsen & von Ins, 2010), and the SDR/SED-WoS links reveal that the by-field variation also exists during the pre-PhD period as shown in Figure 5a. Panel A of Figure 5 shows the probability that people publish at least one article before receiving their PhDs by field of study and source of support. People who received fellowship and/or grant support or were supported as research assistants consistently show higher rates of pre-PhD publishing (Figure 5a). The publishing trajectories post-graduation during the early career period are closely related to pre-PhD publishing and future employment type (Figure 5b). For graduates from the UMETRICS universities, the SED-UMETRICS linkage could add rich data on funding duration and graduate research activities to enable a deeper dive into exploring the implication of how funding support can impact research and future career outcomes.

The above two linkage projects offer a clear example of the ways in which data mosaics anchored in interdependent network organizations can promote dramatic expansions in the value of linked data. IRIS, NCSES, and the Coleridge Initiative together created a new partnership and with it a new set of linkages between two core data assets (UMETRICS and SED) whose integration strengthens each. Because both those individual assets are themselves central nodes in existing linked-data architectures, the connection opens new possibilities by virtue of the ‘indirect’ network connections that can be realized, in this case, through a UMETRICS-SED-SDR-WoS linkage. The common data platform represented by the ADRF and the institutional features of IRIS and the IRIS-NCSES partnership ensure that the value of such ‘extended’ linkages can flow both ways. For instance, ‘closing the loop’ between UMETRICS and WoS via validated SED-SDR linkages adds immediate value to UMETRICS based studies of innovation graduate education and scientific careers *and* creates resources that can support further extensions of UMETRICS that treat this linked subset of data as a training resource for supervised machine learning. At the same time, other data assets that are linked to UMETRICS (for instance US Patent Data, information on vendor and subcontractor organizations, and data on the career and employment outcomes of university research employees) become accessible to integration with SED, SDR, and WoS, though realizing some of those connections will require the addition of new partners to the network.

## 8. General Lessons for Building Network Communities for Data Construction

The days when complete, documented, self-contained data sets are handed off from a single, authoritative source to an anxious but largely passive user community are over. Rather than a system of more or less benevolent data monopolies that respond (or do not) to the needs of distinct, well-defined user groups by fiat, we now face the challenge of building and sustaining federated data systems comprised of many coequal data providers and heterogenous, fluid groups of data users who face different, sometimes contradictory restrictions and requirements while seeking to achieve disparate and sometimes orthogonal goals. Matters are complicated because the skills and capabilities necessary for responsible, effective integration and use of disparate, dynamic, and continually changing data sets are often not housed in any single organization. As a result, the research community must be directly and intimately involved in the work of data production and integration on an ongoing basis. That integration has great potential to inform and improve the work of both groups, if the right synergies can be cultivated.

We argue that those synergies are technical, pertaining to the rigor and quality of linkages across complementary assets; processual, pertaining to the engagement of community and training to build capacity for responsible, inclusive data use; and organizational, pertaining to the governance and institutional arrangements of partnerships themselves. Data mosaics, founded on network forms of organization that promote generalized exchange in the service of many goals, we propose, represent a novel means to sustainably realize the dramatic value offered by diverse, linked-data architectures. They are thus an alternative to the centralized and decentralized models of data sharing that, while valuable, make it more difficult to address the needs of a strategic, responsible, equitable, timely, and above all relevant and valuable strategic data infrastructure.

We present key concepts underpinning this idea and highlight general dangers that might accompany this new approach. The value of such efforts can be realized and the dangers proactively addressed through evolving partnerships that bring together complementary data assets whose linkage provides value to all parties in a process that transparently engages important communities through training opportunities that themselves serve many needs. The detailed description of an exemplary partnership (between NCSES and IRIS), collaborative process (for initial SED-UMETRICS linkages), and initial results offers several key lessons.

First, relationships that can support innovative communities in the design and implementation of data mosaics must maintain a laser focus on responsible use of data that generates both independent and joint value for all participants. In the case of the NCSES-IRIS partnership, reliance on a common data platform (the ADRF), carefully designed and aligned privacy protections, and agreements that allow synergies to be realized through the collaboration of network anchors address the former. Clear sources of value that can be realized for each partner and for their joint goals are essential.

Second, such relationships must be understood to be lasting, evolving through the sharing of expertise, the joint articulation of new goals and expansions that build on momentum from initial ‘small wins’ to deepen and broaden partnerships. Generative relationships of the sort that can create and sustain data mosaics are not arms-length, transactional ‘transfers’ of data; they are living partnerships whose characteristics and features must be allowed to grow and change as skills, familiarity, interdependence, and trust increase. Put simply, relationships that work as the basis for network forms of organization are those that are consistently defined by close, joint work.

For instance, with approval from IRIS’s board of directors, NCSES and IRIS are currently working to complete an expanded partnership agreement that will allow direct use of PII laden UMETRICS data for improved linkage and responsibly streamline mechanisms for research access and use of the integrated data that result. This expanded partnership was made possible by the value demonstrated by the collaborative work described here, which in turn was enabled by the initial use of hashed linking methods. The process of demonstrating value through an initial, even imperfect, effort in order to support later expansions demonstrates a key feature of generative partnerships that can anchor a networked data mosaic, the role that ‘small wins’ play in building momentum and community and participant support for new endeavors (Weick, 1984). Developing strong networks for data integration is a long-term game that must often proceed in stages tied to particular opportunities or community needs. To some extent such partnerships must play out transparently, in public, to enable the value of initial work to be realized.

Third, the value of dyadic relationships such as the one we describe here must be realized through transparent engagement with broader communities. In our case, the primary means for that engagement is training courses that serve at least several manifest purposes. Engaging broad communities of users from different stakeholder groups (federal agencies, university researchers, diverse institutions) in active work with early iterations of collaboratively developed data products provides a sustained mechanism for the partnership to build data infrastructure *with* the relevant communities rather than simply *for* them, as evidenced by improvements to both data and documentation that emerge directly from the classes. Likewise, such courses systematically and proactively address a key danger that can lead to network failure, competency shortfalls that can lead to damaging “screw-ups,” by systematically building the capacity and engagement of partners and community members alike. Finally, such broad engagement and capacity-building represents a key means to ensure transparency, limit opportunism, and in so doing ensure the creation of more equitable, inclusive, and broadly relevant data mosaics.

Growing data mosaics through deepening partnerships between network anchors in the context of training opportunities for broad communities of potential users who can contribute to the process has the potential to generate exponential returns as our model suggests both because of direct linkages between data sets and also because drawing links between central nodes in existing data architectures (SED for NCSES and UMETRICS for IRIS) creates many new ‘indirect’ possibilities for further expansion while deepening the capabilities, familiarity, and trust of both partners. The SDR-WoS linkage presented here offers such a case in point. NCSES’s development of this linkage and its use of SED as a sampling frame for SDR mean that should it be deemed valuable, and that expanding the UMETRICS-SED linkage to bring together measures from WoS and SDR could be much more efficiently and effectively accomplished.

The creation, maintenance, and growth of data mosaics conceptualized in terms of communities engaged in network forms of organization to produce deeply integrated and flexible network data architectures offers a new and, we believe, essential model for realizing dramatic value from linked data. Accomplishing that in complex arenas such as the study of innovation requires both technical and social organizational work that can be facilitated but not controlled by network anchors and partnerships among them. Creating national mosaics within and across substantive domains, which are, in essence, networks of networks with full attention to collective and individual value, interdependence, responsible data use, and equity, requires the ongoing work and engagement of communities. Collective efforts to accomplish such work are novel and necessary to realizing the value of linked data envisioned in today’s policy, administrative, and scientific endeavors.

## Figures and Tables

**Figure 3.**
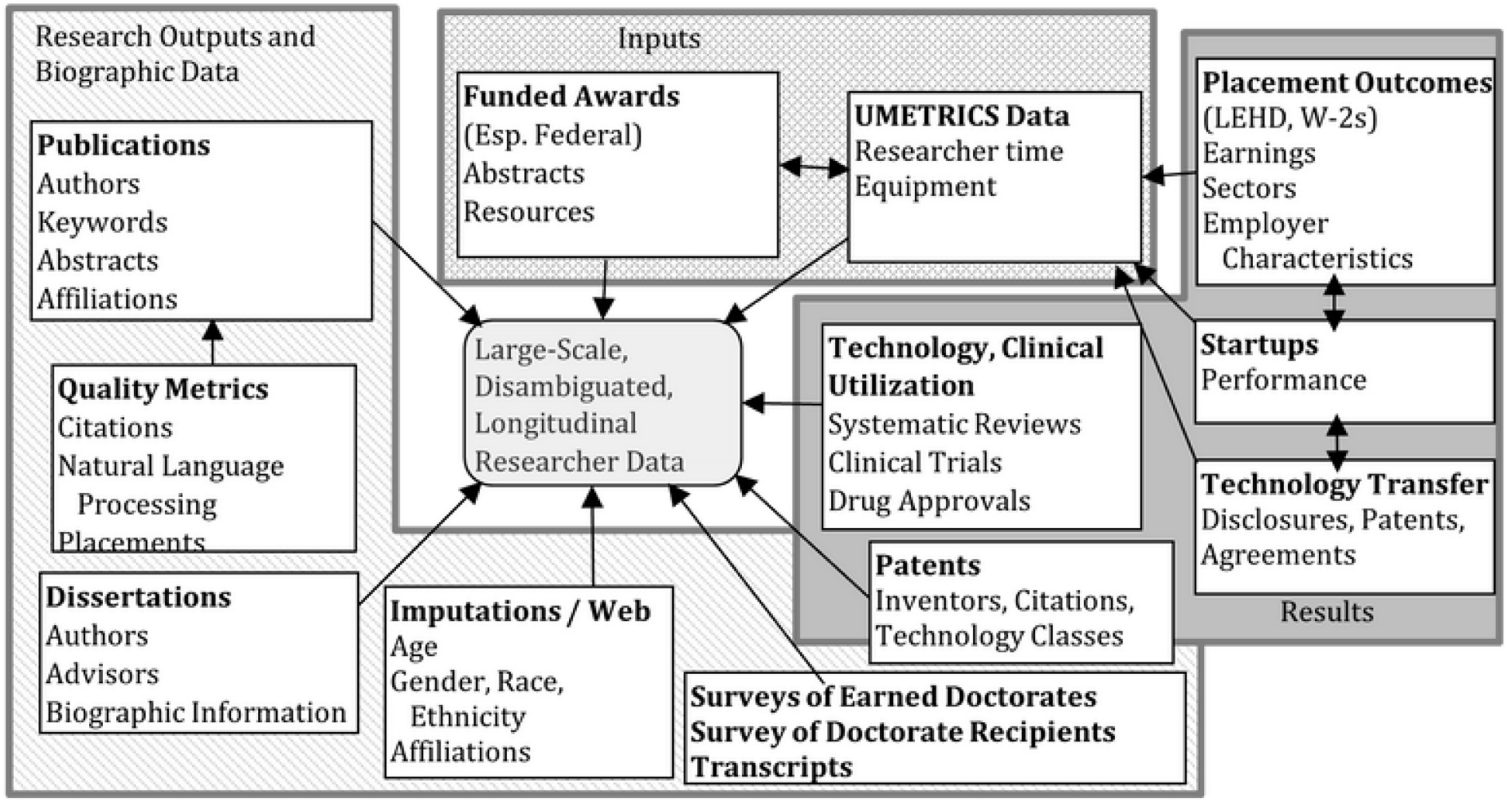
Large-scale disambiguated longitudinal researcher data architecture. Emerging data architecture organized by links. NIH = National Institutes of Health. ORCID = Open Researcher Contributing ID. PIs = Principle Investigators. SED = Survey of Earned Doctorates. UMETRICS = Universities Measuring the EffecTs of Research on Competitiveness, Innovation and Science

## Appendices

### Appendix A: SDR-Web of Science Linkage

Respondents in the 2015 Survey of Doctorates Recipients are linked to authored publications indexed in the Web of Science and appeared during 1990–2017. Two different stages of machine learning approaches were implemented sequentially and illustrated in Figure A1. In stage 1, high-confidence matches were determined by applying a supervised gradient boosting machine learning algorithm (GBM) constructed with 67 features ranging from comparable direct and indirect identifiers to auxiliary variables containing only information from SDR (e.g., race/ethnicity) or Web of Science (WoS) (e.g., name commonality.). The GBM model was trained on a gold standard set, which is a representative random sample stratified on SDR respondent demographics, education, and employment characteristics as well as the frequency of occurrence of a respondent’s name among all publications indexed in the Web of Science, referred to as name commonality. The search for high-confidence matches occurred only within an individual respondent’s Candidate Name Block, a subset of WoS publications selected using a set of rules based on all name variants provided by a respondent (Last name plus First initial, LNFI, blocking) to capture a respondent’s publications efficiently and inclusively and reduce computational load. To mitigate the effects of varying model performance and class imbalance on the overall precision rate, the stage 1 results were structured into subgroups defined by the top five predictors and the model threshold is set at the subgroup level to achieve an overall 95% precision.

In stage 2, the resulting high-confidence publications were used to seed and selectively append additional publications from WoS Author Clusters, an independent methodology that disambiguates the more than 171 million WoS publication records. The goal for stage 2 was to maintain the stage 1 Precision results for each subgroup while augmenting the Recall. Precision and Recall rates were also measured at the subgroup level to provide a transparent understanding of the resulting data set’s strengths and limitations. Subgroups with a high-quality correspondence between survey and publication data resulted in Precision and Recall rates each greater than 90% (51.5% of the SDR-WoS matched-publication data set). The quality of the results decreases as the availability or quality of the meta-data decreases. For example, for those subgroups where the strongly disambiguating variables had no high-quality meta-data correspondence, the results demonstrate a need for improved or alternative methods.

### Appendix B: Loss of Precision When DOB Requirements Removed From Matching Criteria

Each filtering level reduces the stringency of the matching criteria. Steps 3–4 includes matches that do not match on the month of birth as long as the month of birth is missing in at least one data set; steps 5–6 do not require any date of birth information as long as the year of birth is missing in at least one data source. The reduction in stringency increases the likelihood of false positives: incorrectly identifying individuals as matched when they are not the same individual.

The false positive match rate is calculated by matching the entire sample using the step 3 and step 4 criteria but removing the requirement that month of birth had to be missing in one data set. After applying five filters that removed less confident matches, 24,120 matches were identified with month and year of birth available in both data sets; as reported in Table A1, 19,119 (79.27%) had the same month-year of birth while 5,001 (20.73%) had a different month-year of birth. Meaning that if the month-year of birth had been available in both data sets, we estimate that 20.73% of the matches constructed during steps 3–4 would not have been matched using steps 1–2 matching criteria. Using our full matching algorithm there were 126 matches constructed during steps 3–4, as reported in Table A2. Applying our false positive rate, we expect that about 100 of those matches would have the same month-year of birth, while 26 of them would not have the same month-year of birth.

The same approach was applied for steps 5–6 where we do not match on any date of birth information (again removing the requirement that year of birth be missing in at least one data set). After applying five filters that removed less confident matches, the expected precision loss was 23.31%. Using our full matching algorithm, there were 1,035 matches constructed during steps 5–6; applying our estimated false positive rate, we expect that about 794 of those matches would have the same month-year of birth, while 241 of them would have not had the same month-year of birth. While applying the less stringent matching criteria resulted in false positive matches, not applying this criteria would have resulted in far more false negative matches.

**Table A1.** Loss of Precision When DOB Requirements Removed From Matching Criteria

	Steps 3–4	Steps 5–6
Matches with month-year of birth information available	24,120	24,105
Matches with matching month-year of birth	19,119	18,487
Matches with wrong month-year of birth	5,001	5,618
Loss of precision compared to steps 1–2	20.73%	23.31%

*Note.* For column 1, matches were constructed by matching the entire sample using the steps 3–4 criteria but removing the requirement that month of birth had to be missing in one data set. And then applying the 5 filters that were used to identify the most confident matches. After identifying matches with month-year of birth available, the member of matches with matching month-year of birth and with non-matching month-year of birth were identified and a loss of perception compared to steps 1–2 was calculated. The process was repeated using the steps 5–6 criteria and are reported in column 2.

**Table A2.** Match counts by matching step.

Step	Matches	Percent of Matches	Matching Criteria
**1**	8,916	59.57%	Full given name, family name, UMETRICS institution ID, month-year of birth
**2**	4,892	32.68%	First word in given name, family name, UMETRICS institution ID, month-year of birth
**3**	87	0.58%	Full given name, family name, UMETRICS institution ID, year of birth, (month of birth must be missing from SED and/or UMETRICS)
**4**	39	0.26%	First word in given name, family name, UMETRICS institution ID, year of birth, (month of birth must be missing from SED and/or UMETRICS)
**5**	749	5.00%	Full given name, family name, UMETRICS institution ID, (year of birth must be missing from SED and/or UMETRICS)
**6**	286	1.91%	First word in given name, family name, UMETRICS institution ID, (year of birth must be missing from SED and/or UMETRICS)
**Total**	14,969	100%	

*Note.* This table reports the match counts by matching step, percent of matches that were made during each step. And the matching criteria for each step. This table represents the matches after applying the filter to identify the most confident matches. SED = Survey of Earned Doctorates.
